# A new lower limb portable exoskeleton for gait assistance in neurological patients: a proof of concept study

**DOI:** 10.1186/s12984-020-00690-6

**Published:** 2020-05-06

**Authors:** G. Puyuelo-Quintana, R. Cano-de-la-Cuerda, A. Plaza-Flores, E. Garces-Castellote, D. Sanz-Merodio, A. Goñi-Arana, J. Marín-Ojea, E. García-Armada

**Affiliations:** 1 Escuela Internacional de Doctorado, Rey Juan Carlos University, Madrid, Spain; 2 MarsiBionics S.L., Madrid, Spain; 3 Department of Physiotherapy, Occupational Therapy, Rehabilitation and Physical Medicine, Faculty of Health Sciences, Rey Juan Carlos University, Avda. Atenas s/n, 28922 Madrid, Spain; 4 Centre of Automation and Robotics, CSIC-UPM, Madrid, Spain; 5 Escuela de Doctorado, Alcalá University, Madrid, Spain; 6 Aita-Menni Hospital, Bilbao, Spain

**Keywords:** Exoskeletons, Feasibility, Gait, Multiple sclerosis, Safety, Stroke

## Abstract

**Background:**

Few portable exoskeletons following the assist-as-needed concept have been developed for patients with neurological disorders. Thus, the main objectives of this proof-of-concept study were 1) to explore the safety and feasibility of an exoskeleton for gait rehabilitation in stroke and multiple sclerosis patients, 2) to test different algorithms for gait assistance and measure the resulting gait changes and 3) to evaluate the user’s perception of the device.

**Methods:**

A cross-sectional study was conducted. Five patients were recruited (4 patients with stroke and 1 with multiple sclerosis). A robotic, one-degree-of-freedom, portable lower limb exoskeleton known as the Marsi Active Knee (MAK) was designed. Three control modes (the Zero Force Control mode, Mode 1 and Mode 3) were implemented. Spatiotemporal gait parameters were measured by the 10-m walking test (10MWT), the Gait Assessment and Intervention Tool (G.A.I.T.) and Tinetti Performance Oriented Mobility Assessment (gait subscale) before and after the trials. A modified QUEST 2.0 questionnaire was administered to determine each participant’s opinion about the exoskeleton. The data acquired by the MAK sensors were normalized to a gait cycle, and adverse effects were recorded.

**Results:**

The MAK exoskeleton was used successfully without any adverse effects. Better outcomes were obtained in the 10MWT and G.A.I.T. when Mode 3 was applied compared with not wearing the device at all. In 2 participants, Mode 3 worsened the results. Additionally, Mode 3 seemed to improve the 10MWT and G.A.I.T. outcomes to a greater extent than Mode 1. The overall score for the user perception of the device was 2.8 ± 0.4 95% CI.

**Conclusions:**

The MAK exoskeleton seems to afford positive preliminary results regarding safety, feasibility, and user acceptance. The efficacy of the MAK should be studied in future studies, and more advanced improvements in safety must be implemented.

## Background

In 2015, neurological disorders accounted for 16.8% of the total deaths worldwide and 10.2% of the global disability-adjusted life-years (DALYs) [1]. These numbers have increased since 1990 due to growing size of the population and aging, and they are expected to continue to increase. By 2030, it is estimated that the population affected by neurological diseases will include as many as 1.136 million people [2]. In Spain, between 6.7–7.5 million people are affected by neurological diseases [3]. The total direct and indirect cost related to neurological diseases was 10.9 million euros in 2004 in this country [3, 4].

Neurological diseases cause functional disturbances, including gait disabilities, that affect patients’ ability to perform activities of daily living [1]. Between 50 and 60% of patients with stroke still have some degree of motor impairment after a conventional rehabilitation period [5]. In multiple sclerosis (MS) patients, gait impairment is a major contributor to social, personal and economic burdens [6]. Thus, gait impairment is one of the main problems in patients with stroke or MS [7, 8].

Due to the extent that gait impairment affects patients, gait rehabilitation is considered a key aspect of physical rehabilitation [9–14]. Currently, there is a growing interest in determining which characteristics of training should be involve in gait rehabilitation, as therapies are currently based on repetitive and intensive training and functional and feedback-based interventions [15–17]. These characteristics are aligned with the use of exoskeletons in gait rehabilitation. In recent years, this technology has been widely used in stroke and MS studies [18–24].

**Fig. 1:**
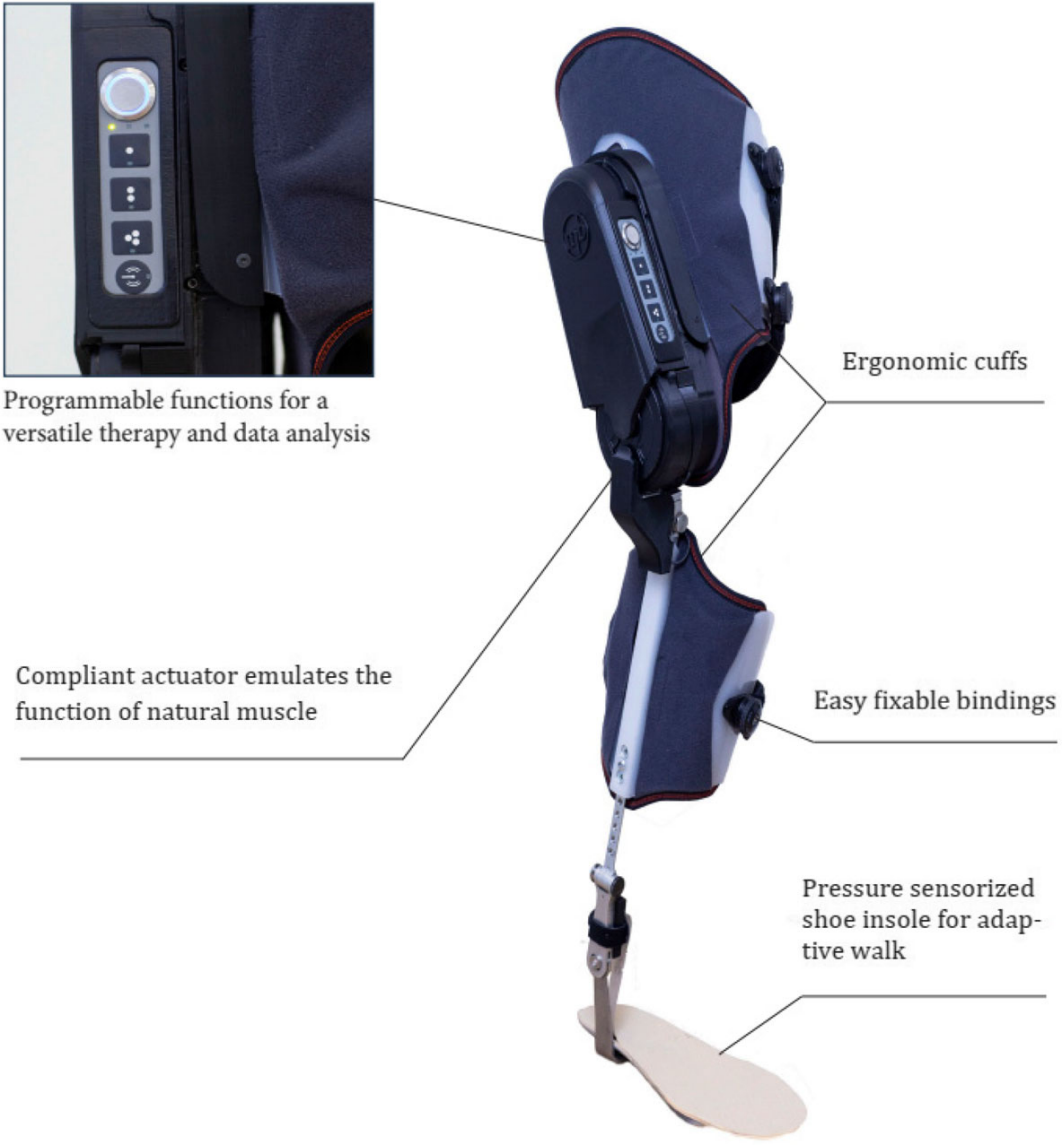
Marsi Active Knee (MAK) exoskeleton, by Marsi Bionics

To the best of our knowledge, few portable exoskeletons that are lightweight and have the capability to execute or modify gait assistance algorithms have been developed, and a high degree of customization can be allowed by following the *assist-as-needed* concept [25] for gait assistance in stroke and MS patients. The exoskeleton evaluated in this study is a single-limb exoskeleton with actuation at the knee level (Fig. 1). Thus, the main objectives of this study were 1) to explore the safety and feasibility of the exoskeleton developed by the research team for gait rehabilitation in stroke and MS patients as a proof of concept, 2) to test different algorithms for gait assistance and measure the resulting gait changes and 3) to evaluate the user’s perception of the device.

## Methods

### Design

This cross-sectional study was conducted at the Rehabilitation Service of the Aita Menni Hospital in Bilbao (Spain). Informed consent was obtained from the patients prior to their inclusion in the study, which was conducted in accordance with the Declaration of Helsinki. All assessments were performed by the investigator GPQ (PT).

### Participants

The inclusion criteria were as follows: an age between 18 and 75 years, a diagnosis of stroke that was confirmed by computed tomography or magnetic resonance imaging [26] or of MS that was made according to the McDonald criteria [27], muscle spasticity in the lower limbs corresponding to a score of fewer than 3 points on the Modified Ashworth Scale (MAS) [28], the ability to walk 10 m (with assistance, if needed), a Functional Ambulation Category (FAC) score of between 1 and 3, and Mini Mental State Examination score of higher than 24 points [29].

The exclusion criteria were the presence of any other neurological, vestibular, orthopedic or systemic alteration that could affect the individual’s gait ability, a body weight of more than 100 kg, and a height of less than 1.5 m or more than 1.90 m (to meet the size criteria to use the exoskeleton). In addition, patients who had joint contractures in the lower limbs or underwent surgical interventions in the past 2 years were excluded.

### Main description of the exoskeleton

The Marsi Active Knee (MAK) exoskeleton is a robotic knee orthosis that provides walking assistance to patients with weakness in the lower limbs under the supervision of qualified rehabilitation staff (Fig. 1). Using this device, the motor action of the user is complemented in the sagittal plane of the knee with a system that augments the user’s strength and supports the user. The system is able to mobilize the user’s knee and provide as much cushioning as needed. When this proof-of-concept study was designed, the MAK exoskeleton presented a technology readiness level (TRL) of 6, and during the course of this study, whether a TRL of 7 could be reached was determined [30, 31].

The performance of the orthosis is based on the microprocessor control of an electric motor that supplies sufficient power to assist knee flexion and extension during walking. For patients with gait impairments, this device provides additional support and mobilization of the knee during the swing phase and, when necessary, prevents involuntary knee flexion. It may improve the safety and independence of the user and allow the user to execute more natural and symmetrical movements during gait. The user and the therapists can select the modes of operation using the buttons on the device surface. The patients were monitored and evaluated in real time according to the data storage and recording capacities for analysis.

The weight of the device is 2.8 kg, the maximum walking speed allowed is 1 m/s, and under normal conditions, the battery lifetime is approximately 4 h of continuous use.

### Mechanical design of the exoskeleton

The MAK exoskeleton can be considered, from a mechanical point of view, a lower limb exoskeleton for the knee joint with one active degree of freedom (DOF) (Fig. 1).

The action of the device is based on the actuation of an electric motor that, in combination with the gear, provides the expected torque and speed. The actuator also has a spring ensemble that absorbs shock, vibrations and the joint dynamics of the user, thus turning the actuator into a rotary elastic actuator [32]. The components of the MAK include the control module, structure, orthopedic fastenings, battery and foot insoles:

Control module: The part of the system that generates movement; it consists of the motor, elastic elements, the main electronics and the entire casing that surrounds it.

Structure: The element that transmits the mechanical energy provided by the control module. It includes the upper structure (thigh) and lower structure (calf). The structure is adjustable in height and can fit users with heights ranging from 1.50 m to 1.90 m.

Orthopedic fastenings: The parts of the device that fix the orthosis to the patient and adapt the shape of the structure to the user’s leg so that the transmission of energy does not cause any injuries. The fastenings have thigh, calf and static ankle-foot orthosis (AFO) components.

Battery: It is an independent module that the user can wear on the hip with a belt. It is connected to the control module and supplies power to the device.

Foot insoles: The foot insoles are parts of the device that measure the pressure registered at 8 different places on the foot. There are 2 parts on the heel of the foot (named the Interior heel and Exterior heel), 1 at the middle of the medial longitudinal arch (named Arch), 3 at the metatarsal heads (first, third and fifth metatarsal head of the toes, named Met 1, Met 3 and Met 5, respectively), another at the tip of the second to fifth distal phalanx of the toes (named Toes), and 1 at the distal phalanx of the first toe. The shoe insole sizes ranged from 37 to 45 cm, consistent with the European convention for shoe sizes.

### Control design of the exoskeleton

The MAK exoskeleton includes a series of sensors that allow information regarding the state of the equipment and the actions of the user to be collected. Considering the scope of this work, the sensors of interest are as follows (Table 1):

1. The absolute position encoder in the actuator assembly, which measures the angular position of the knee.
2. The insoles with embedded sensors (previously mentioned). The user wear shoe insoles embedded with several sensors in both shoes. The insole on the non-affected side communicates with the MAK through a wired RS-485 connection.

**Table 1:** Sensors in the exoskeleton and collected data

Measurements	Unit	Number of sensors	Sensitivity	Sample Rate	Placement	Minimum/Maximum value:
Angular position	degrees	1	0.088°	0.5 ms	Motor	-5°/115°
Shoe insole pressure sensors	mbar	8	6 mbar	2.5 ms	Shoe insole	7 mbar/ 3000 mbar

Sensor description of the MAK device

**Fig. 2:**
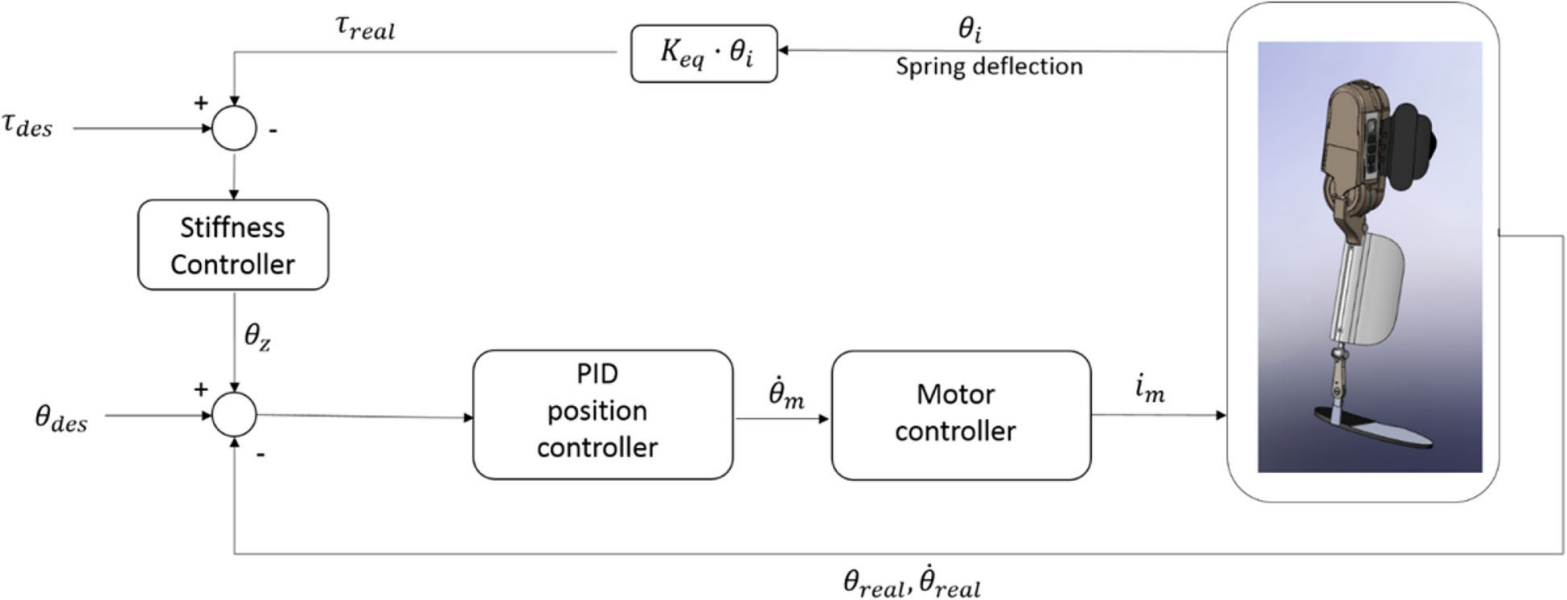
Control scheme of the MAK device

Since the MAK device accurately measures the angular position and the force exerted on the joint, it is therefore capable of sending position/speed and force/impedance control commands. Figure 2 shows the control scheme implemented in the device.

**Fig. 3:**
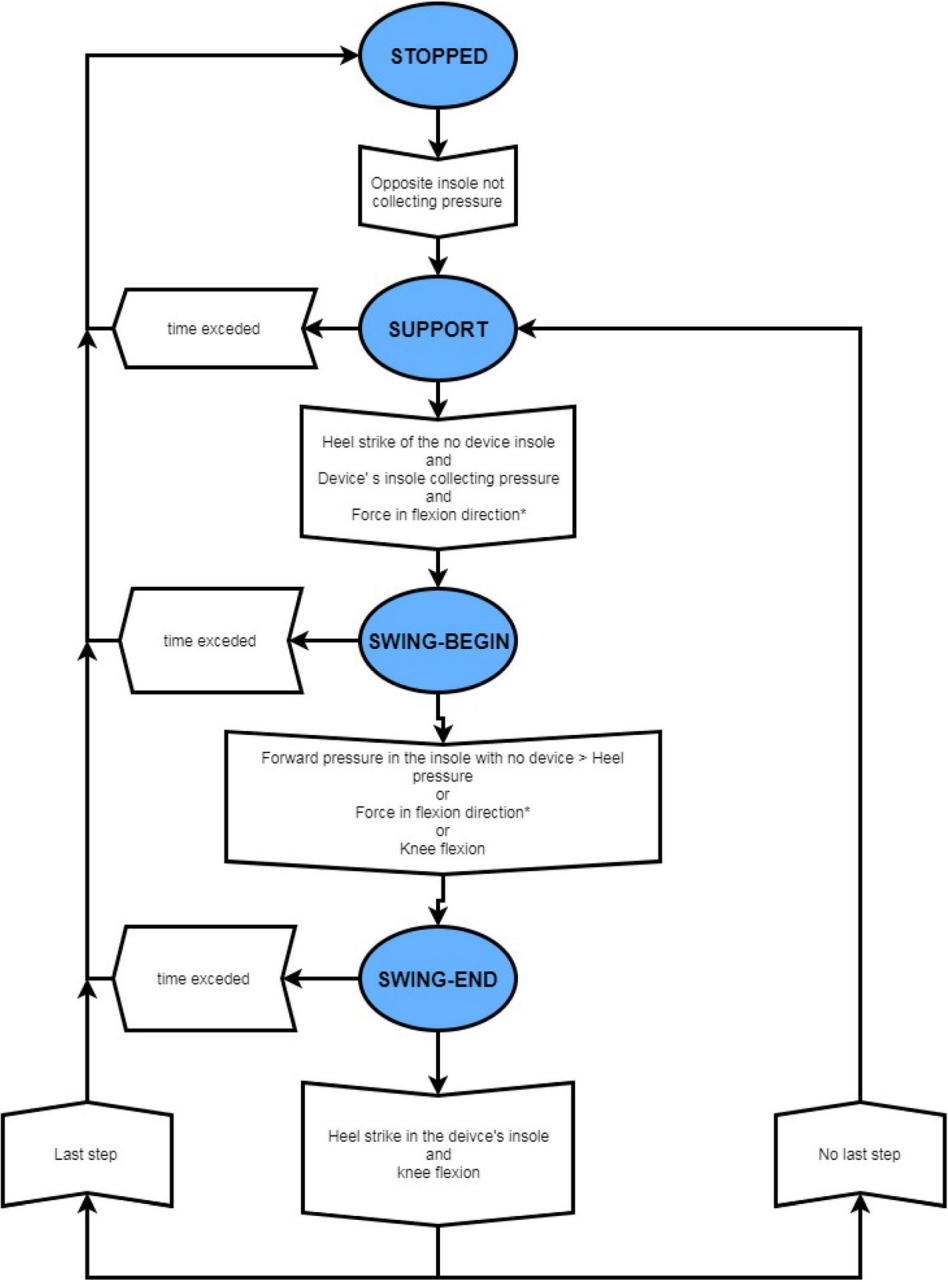
Control scheme of the step decision. Control scheme of the MAK device. Opposite foot: foot from the leg where the MAK device is not attached. *: Only applies for M1

Based on this general control scheme, three control modes (Zero Force Control mode, Mode 1 and Mode 3) have been implemented in the sessions:

1. Zero force control mode (F0): In this mode, zero rigidity is established in the general control scheme. Therefore, when this mode of action is employed, the exoskeleton will follow the user’s intentions without interfering or assisting. This mode was developed by following the principles of a “transparent exoskeleton” mode of actuation and was used to determine how the device affects the user’s gait.
2. Modes 1 (M1) and 3 (M3) are active-assistance modes. According to measurements from the sensors presented in the previous sections, the gait phase of the patient can be detected. The established control scheme differs depending on the phase of gait detected by the device. Figure 3 shows the state machine implemented in the device and the triggers required for each state transition. If a foot insole is disconnected, the MAK saves the most recent data recorded and uses it throughout the remainder of the session in which the device is used. In the support phase, the control system uses position control, the knee is extended, and a high stiffness value is used, which can be changed according to the strength of the user so that the user can support the weight of his or her own body with the aid of the device. In the swing phase, speed control with a modifiable rigidity is also used to allow the user to apply forces in this phase.

In the M1 control mode, the device detects the user’s movement to change the phase in the state machine and adapts to it within the “*assist-as-needed*” paradigm. M1 is used as a trigger to change the machine state based on the following data: the knee joint angle, pressure at the shoe insole and force at the knee joint.

In M3, the device maintains a fixed continuous mode in which the device transitions between states in the state machine automatically. In this mode, the level of assistance remains constant. The velocity is dependent on the duration of the swing phase of the other leg. The trigger used by M3 to transition between states in the state machine is a function of the knee joint angle and pressure recorded from the shoe insole.

### Trial procedure

A proof-of-concept study was conducted to test the MAK exoskeleton with stroke and MS patients. Each participant performed a single session, during which they were asked to perform one trial of the 10-m walking test (10MWT) in a hallway at a comfortable speed [33]. During all the sessions, a rehabilitation clinician closely followed the participant but did not touch them to ensure their safety. These tests were first carried out without the exoskeleton and were later on carried out with the exoskeleton worn on the affected limb. When the exoskeleton was used, the 3 possible exoskeleton actuation modes were employed. The settings of the device were changed by the investigator APF at the end of each 10MWT. The trials were performed in the following order:

1. 10MWT without the exoskeleton.
2. 10MWT with the exoskeleton under M1 mode.
3. 10MWT with the exoskeleton under F0 mode.
4. 10MWT with the exoskeleton under M3 mode.

Between each test, a rest period of 5 min was provided to mitigate the effects of fatigue. While the participant were performing the test, four video cameras (Nikon D3100, Tokyo, Japan) were used to record the participant’s gait: two were placed at each side, another was placed in front of the participant and the last one was placed behind the participant at the start of the walking path.

All participants had some experience using the MAK exoskeleton. Before this study, each participant used the device for 5 sessions lasting 50 min each. The experience was important for adapting the device to the user’s lower limb. The ergonomic design of the device was developed during these previous sessions.

### Measurements

The safety and feasibility of the device were assessed by the record of the adverse effects and malfunction events during the trials. The adverse effects included any damage or unexpected outcomes during the trials, such as skin changes, bone fractures or falls.

Spatiotemporal gait parameters were measured by the 10MWT, the Gait Assessment and Intervention Tool (G.A.I.T.) and Tinetti Performance Oriented Mobility Assessment (gait subscale) before and after the trials. A modified QUEST 2.0 questionnaire (Table 2) was administered to determine the participants’ opinions about the exoskeleton. The data acquired by the sensors placed on the exoskeleton were normalized to a gait cycle. Finally, during the use of the MAK exoskeleton, adverse effects were recorded.

**Table 2:**
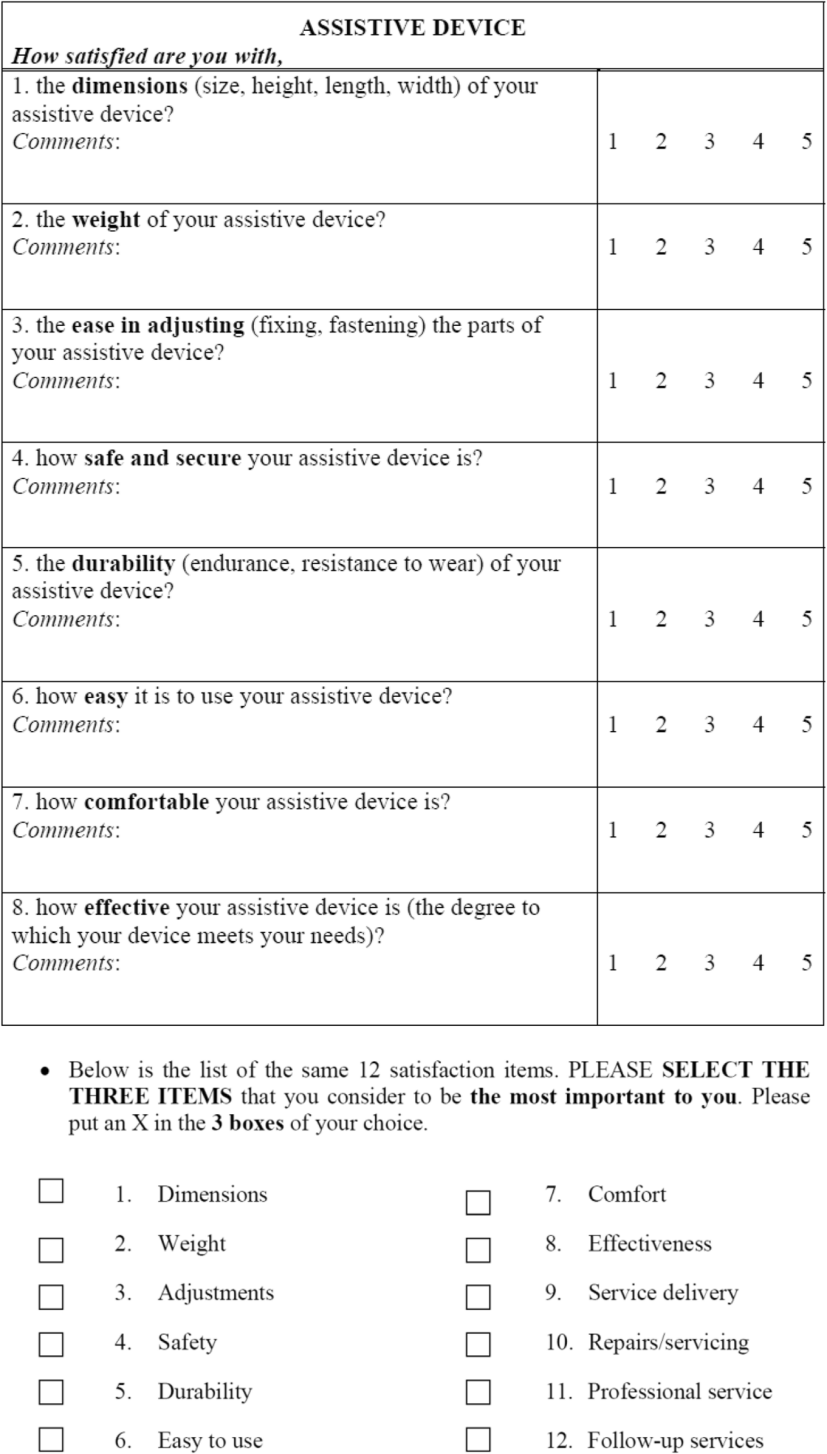
Modified QUEST 2.0 questionnaire

The QUEST 2.0 was modified in order to fit with the aims of the study. This modification consisted of not assessing the Services domain and the items in the multiple choice question regarding the Services of the exoskeleton provider

The 10MWT was performed as follows: the start of the 10 m course was marked with tape on the floor in the middle of a 12 m-long corridor [33]. The participants positioned their toes behind the start line and were instructed to walk at their comfortable speed and continue walking down the corridor until they were told to stop. The stop command was given approximately 1 m past the finish line so that participant did not decelerate until after passing the 10 m mark.

The G.A.I.T [34]. is an observational gait assessment tool composed of 31 items divided into three sections: 4 items on the upper limb and trunk, 14 on the lower limb and trunk during the stance phase, and 13 on the swing phase. All of the items are rated on a 4-point scale (from 0 to 3), where 0 corresponds to normal behavior and 3 corresponds to the maximal deviation. The highest score possible for the scale is 62. Its psychometric properties have been analyzed, showing excellent intra- and interrater reliability, moderate correlation with the data obtained by a tridimensional motion capture system, and sensitivity to changes after a gait training program in people with stroke [35]. The Spanish cross-cultural and translated version for stroke and MS was used in this study [36].

The Tinetti Performance Oriented Mobility Assessment (gait subscale) was used. Although this scale was originally developed to assess gait disturbances in geriatric patients [37], it has also been used and studied in patients with neurological disorders, especially in those with stroke, MS and Parkinson’s disease [38–40]. It comprises two subscales for gait and balance. The gait subscale consists of 10 items scored from 0 to 1 or 2. The maximal score of the gait subscale is 162. The maximal score of the full scale is 28.

The MAK is able to record data from the wearer, such as the knee joint position, shoe insole pressure at each sensor and the center of pressure (CoP). The data were recorded at a sampling rate of 100 Hz.

The joint angles of the knees were determined by the data from the encoder embedded in the actuator assembly. For comparison purposes, the mean and standard deviation of the filtered (4th order low-pass Butterworth filter with a cutoff frequency of 1 Hz) knee angle was computed for each subject and operating mode.

The data used to calculate the pressure of the feet were recorded at 8 points, as previously mentioned in the *Mechanical design of the exoskeleton* section, from both of the user’s feet. From these data, the CoP trajectory was defined by the location of the sensor under the foot. In this case, the reference point of the coordinate system was located on the outer edge of the heel. To estimate the CoP of a foot, the pressures measured by all the sensors weighted by their corresponding coordinates were summed and then divided by the overall pressure at each time instant, as shown in Eq.1 [41]:

$$ CoP=\frac{\sum_{s=1}^n{p}_{\overset{s}{n}}\cdot \left({x}_{s,}{y}_s\right)}{\sum_{s=1}{p}_s} $$

where *n* is the number of sensors in the insole, *p*_*s*_ is the pressure measured by sensor *s*, and (*x*_*s*_, *y*_*s*_) are the spatial coordinates of sensor *s* in the coordinate system defined by the location of the sensors within the insole. For each subject and mode of operation, the average CoP of all strides was computed.

A gait cycle was defined as a cycle from heel strike to the following ipsilateral heel strike, which consisted of two phases: 1) stance phase, the period of time during which the foot remained on the ground, and 2) swing phase, when the foot was off of the ground and moved forwards. A sensor was considered active when its measurement exceeded a given threshold value to avoid false positives. A foot was considered in the stance phase of a gait cycle when at least one of the pressure sensors in the ipsilateral insole was active; otherwise, the foot was considered in the swing phase of the gait cycle. Heel strikes were defined as the instants in which the foot changed from being in the swing phase to the stance phase. Each trial was split into complete strides for both the healthy and the affected leg separately using the identified heel strike events.

A modified QUEST 2.0 questionnaire was administered after the exoskeleton trials. The Quebec user evaluation of satisfaction with assistive technology test (QUEST 2.0) was originally developed by Demers and coworkers and assesses a person’s positive or negative opinion of the dimensions of an assistive device that are influenced by one’s expectations, perceptions, attitudes, and personal values [42]. The test assesses how satisfied a person feels with some specific features of the assistive device, as well as certain characteristics of the services delivered by it. QUEST 2.0 essentially consists of an 8-item device domain and a 4-item service domain and is one of the few standardized instruments that was designed to measure user satisfaction with a broad range of assistive technology devices. Each item is rated on a 5-point ordinal scale graded from 1 (not satisfied at all) to 5 (very satisfied). For the purposes of this study, a modification of the QUEST 2.0 scale was proposed by including only the items from the assistive device section (Table 2). Additionally, the overall score and that for the multiple-choice item were determined. Due to the specific nature of the trial, only scores ranging from 1 to 8 were allowed for the multiple choice item.

### Statistical analysis

To assess the differences between not wearing the exoskeleton and each mode of actuation provided by the MAK exoskeleton (F0, M1 and M3), the following comparisons were performed:

1. Not wearing the MAK vs wearing the MAK using the F0 control (NoMAK vs F0).
2. Not wearing the MAK vs wearing the MAK using M1 control (NoMAK vs M1).
3. Not wearing the MAK vs wearing the MAK using the M3 control (NoMAK vs M3).
4. Wearing MAK using the M1 control vs wearing the MAK using the M3 control (M1 vs M3).

Due to the expected small sample size and number of sessions (since this study is a proof-of-concept study), only descriptive analysis was performed. The quantitative data are expressed as the percentage of change (if the variable is continuous) or as the absolute score (if the variable is discrete). Data gathered from the MAK are expressed as the median ± standard deviation, normalized by a gait cycle. The data related to the QUEST. 2.0 Questionnaire are expressed as the average ± 95% confidence interval (95% CI). The statistical calculations were conducted in SPSS version 25.0 (IBM, Armonk, NY).

## Result

Five participants were recruited for this study. Participants 1 to 4 were diagnosed with stroke, and participant 5 was diagnosed with MS. The demographic and anthropometric data of the participants are shown in Table 3. Participant 1 was not able to perform the trial using actuation protocol M3 due to fatigue. For the analysis regarding the M3 actuation protocol, the data from the remaining 4 participants were used. The results are divided into the different aims of the present study:

**Table 3:** Socio-demographic and anthropometrics data of the participants

	Patient 1	Patient 2	Patient 3	Patient 4	Patient 5	Median	SD
Gender	Male	Male	Female	Male	Male	–	–
Affected side	Left	Left	Right	Left	Left	–	–
Diagnosis	Stroke	Stroke	Stroke cerebellum	Stroke	Multiple Sclerosis	–	–
Height (cm)	171	173	175	188	169	175.2	7.5
Weight (kg)	82.5	74	65	84	68	74.7	8.5
Months since stroke onset	84	152	21	18	–	69	63
Tinetti scale	14	15	22	14	22	17.4	4.2
FAC	1	2	3	2	3	2.2	0.8
Assistive device used to perform gait	Quadripod cane	Cane	None	Cane	Cane		
MAS Knee Extensors	1	0	1	2	1	1	0.7
MAS Knee Flexors	1	0	1	2	1	1	0.7

Socio-demographic and anthropometrics data of the participants. *Tinetti scale* Tinetti Performance Oriented Mobility Scale, *FAC* Functional Ambulation Categories, *LL* Lower Limb, *MAS* Modified Ashworth Scale

### Safety and feasibility

No adverse effects were reported, but an incident was recorded during the application of M1 with the MAK with participant 4 because the user lost balance once; there were no major complications (no falls were recorded). Nevertheless, the participant was able to continue and finish the trial and the 10MWT in M1. Additionally, during the trials with participant 4, the shoe insole on the contralateral side, the side on which the MAK was not worn, became accidentally disconnected. As a result, the MAK was unable to collect data from this participant, and the M1 actuation protocol did not provide the expected assistance but instead a limited one (as explained in the *Control design of the exoskeleton* section). These two events are likely to be related. The exoskeleton successfully collected the data for the rest of the variables related to the knee joint angle, pressure at each insole sensor and CoP for all participants.

### Gait assistance outcomes

#### 10MWT

Comparisons between the different modes of actuation of the MAK while performing the10MWT are shown in Table 4. Regarding the comparison between not using the MAK versus the F0 actuation protocol, only participant 4 reported an increase in gait speed (13.44% higher speed) using the F0. On the other hand, the rest of the participants decreased their velocity (from 2.71 to 26.71% less) while using the F0 mode. The analysis of the performance when not wearing the device and using the M1 actuation protocol showed that only participant 2 reported an increase in gait velocity (12.29% more) when the M1 was tested. Nevertheless, the rest of the participants lowered their velocity (from 1.37 to 32.53%) when M1 was applied compared to when they were not wearing the device. The comparison between not using the device and applying the M3 actuation protocol revealed that participants 2 and 4 experienced an increase in velocity (24.56 and 24.09%, respectively) while M3 was used. Participants 3 and 5 reduced their speed with M3 (2.34 and 17.28%, respectively). The results comparing the M1 and M3 actuation protocols show that all participants increased their gait velocity when the M3 actuation protocol was applied (from 10.72 to 25.12%).

**Table 4:** 10MWT scores regarding different MAK’s Actuation Protocols

10MWT scores (m/s)	Participant 1	Participant 2	Participant 3	Participant 4	Participant 5
NoMAKvsF0	26,71%	2,71%	10,90%	−13,44%	24,42%
NoMAKvsM1	18,89%	−12,29%	32,53%	1,37%	31,36%
NoMAKvsM3	–	−24,56%	2,34%	− 24,09%	17,28%
M1vsM3	–	−13,99%	−22,78%	−25,12%	− 10,72%

Results obtained at the 10MWT. The reported data are expressed as percentage of change. Velocity (m/s): meters/second. NoMAK: participants not wearing the exoskeleton; F0: the exoskeleton actuation protocol was the force 0 mode; M1: the actuation protocol of the exoskeleton was the Mode 1; M3: the actuation protocol of the exoskeleton was the Mode 3

#### G.a.i.t

**Fig. 4:**
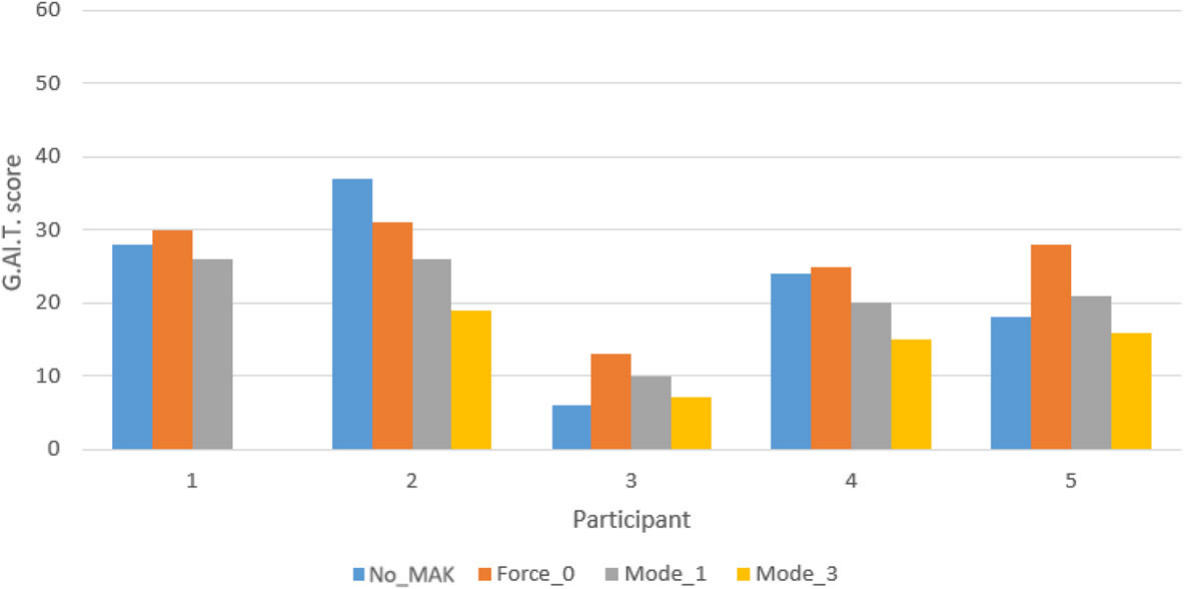
G.A.I.T. scores in different exoskeleton protocol actuations. G.A.I.T. scores in different exoskeleton protocol actuations. Numbers 1-5 at the bottom of the bars represent the patient’s number. No_Exo, the participant was not wearing the exoskeleton; Force_0, the exoskeleton actuation protocol was the force 0 mode; Mode_1, the actuation protocol of the exoskeleton was the Mode 1; Mode_3, the actuation protocol of the exoskeleton was the Mode 3

The absolute scores obtained for the G.A.I.T. are shown in Fig. 4. The differences between the modes of actuation of the exoskeleton are summarized in Table 5.

**Table 5:** Tinetti Performance Oriented Mobility Assessment (gait subscale) scores

Tinetti	Participant Number				
	1	2	3	4	5
**NoMAK vs F0**	0	0	1	− 1	2
**NoMAK vs M1**	−1	0	1	−2	0
**NoMAK vs M3**	–	−3	−1	−1	1
**M1 vs M3**	–	−3	−2	1	1

Tinetti Performance Oriented Mobility Assessment gait subscale scores. NoMAK: participants not wearing the exoskeleton; F0: the exoskeleton actuation protocol was the force 0 mode; M1: the actuation protocol of the exoskeleton was the Mode 1; M3: the actuation protocol of the exoskeleton was the Mode 3

When comparing not wearing the device with the F0 actuation protocol, only participant 2 obtained a better score with F0 (a decrease by 6 points). The rest of the participants had a worse score with F0 (from 1 to 10 points). Regarding the M1 actuation protocol compared to not wearing the device, participants 3 and 5 had worse scores (4 and 3 points, respectively) while using M1. The rest of the participants had improved gait performance (from 2 to 11 points) using M1. When not wearing the device was compared with the M3 actuation protocol, only participant 3 obtained a lower score with M3 (1 point less). The rest of the participants reported a better score (from 2 to 18 points) with M3. When the M1 and M3 actuation modes were compared, a better score was observed for all the participants when M3 was applied (the scores improved from 3 to 7 points).

#### Tinetti performance oriented mobility assessment (gait subscale)

The results of this subscale are shown in Table 5. Even though this subscale has not been validated, the data it provides may be used to When not wearing the MAK and F0 were compared, there were no differences between the modes for participants 1 and 2, participant 4 had a worse score (1 point lower), and participants 3 and 5 received a higher score (1 and 2 points higher, respectively) when F0 was employed. Regarding the difference between not wearing the MAK and M1, participants 2 and 5 did not show any differences (the scores differed by 0 points) between the two conditions. Instead, participant 1 and 4 obtained worse results on the scale (1 and 2 points lower, respectively) using M1, but participant 3 obtained a better score (1 point higher) using this actuation protocol. M3, compared to not wearing the MAK, led to worse scores in participants 2, 3 and 4 (a decrease in the score by 1 to 3 points). Nevertheless, participant 5 obtained a better score (1 point higher) when M3 was employed. Regarding the differences between M1 and M3, participants 4 and 5 obtained a better score (1 point higher in both cases) when using M3. However, participants 2 and 3 obtained a worse result (3 and 2 points lower, respectively) using M3.

**Fig. 5:**
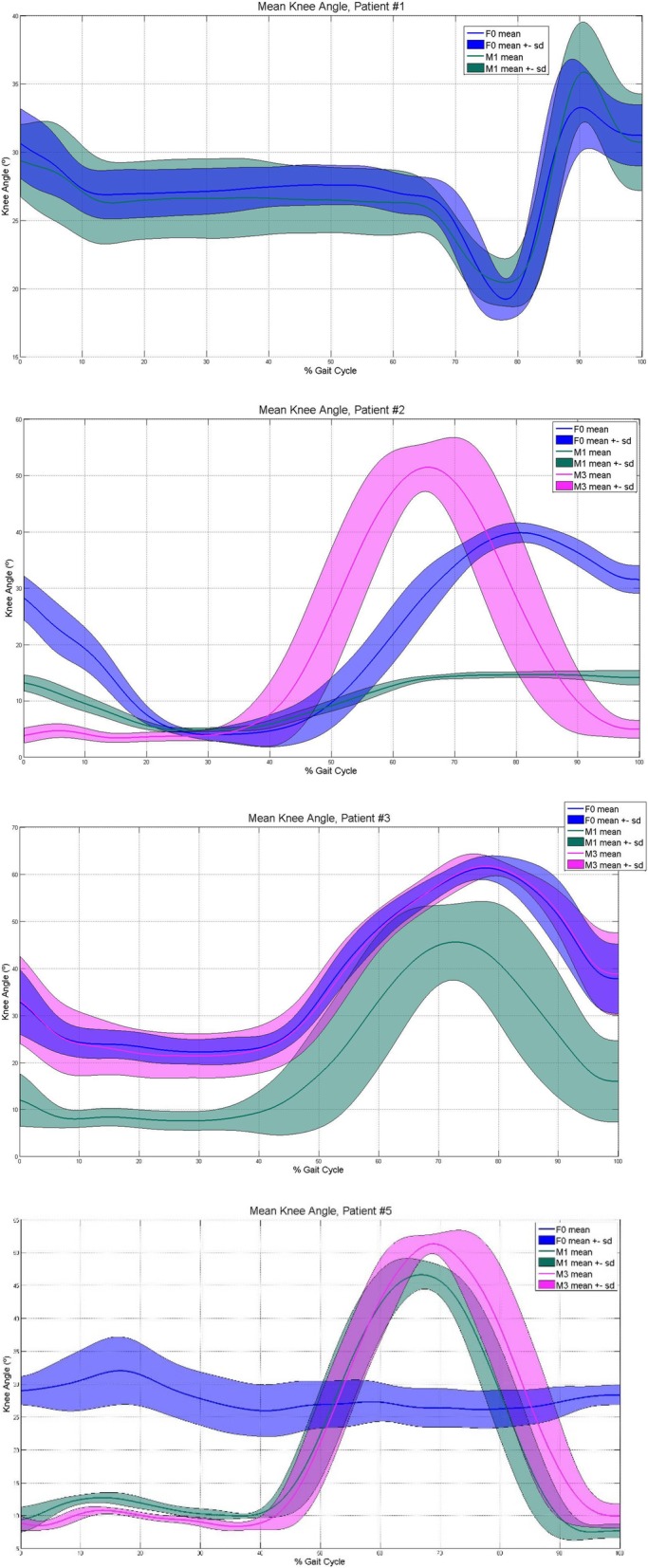
Knee joint angle gathered by the MAK. Knee joint angle gathered by the MAK. F0: F0 actuation protocol; M1: M1 actuation protocol; M3: M3 actuation protocol. Blue color represents F0, green color is related to M1 and pink to M3. A solid line means the median, and the shaded area the standard deviation, given each actuation protocol

**Fig. 6:**
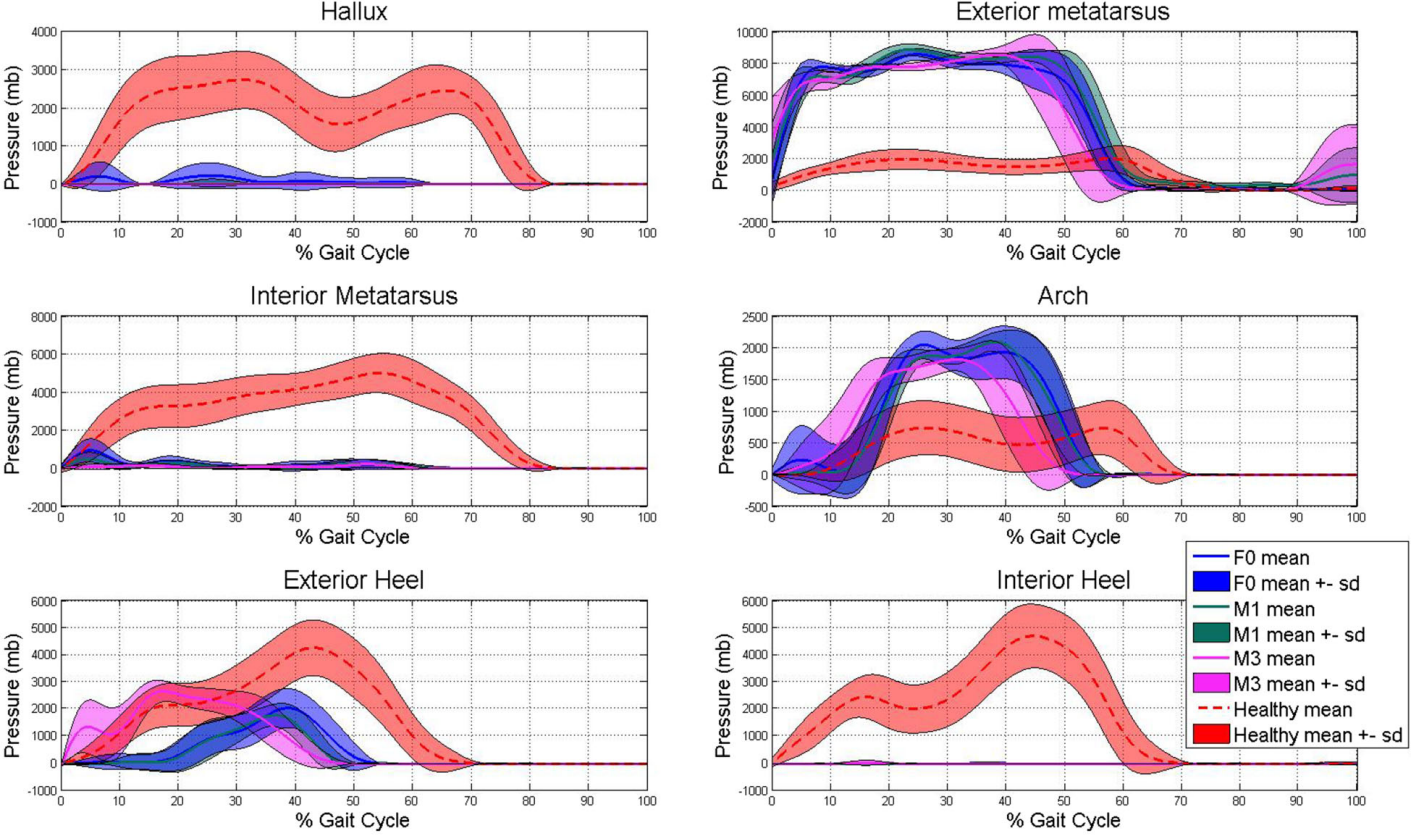
Pressure collected by each sensor in the shoe insole. Pressure collected at each sensor in the shoe insole. F0 actuation protocol; M1: M1 actuation protocol; M3: M3 actuation protocol. Blue color represents F0, green color is related to M1, the pink color to M3 and red color is related to the data collected by the shoe insole placed at the foot where there was no MAK. A solid line means the median, and the shaded area the standard deviation, given each actuation protocol

**Fig. 7:**
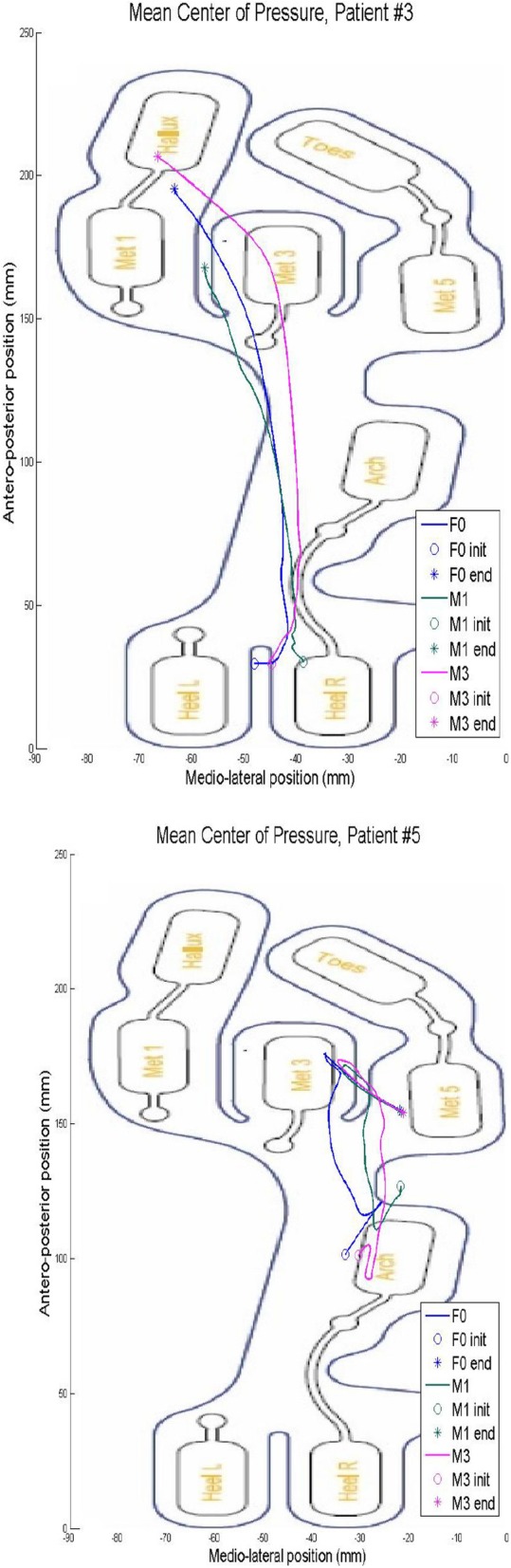
CoP collected in the shoe insole at the foot where the MAK was placed

The exoskeleton MAK collected data on the knee joint angle (Fig. 5), pressure at the sensors in the shoe insoles (Fig. 6) and the CoP (Fig. 7). The data were normalized to a gait cycle according to all the heel strike events detected in the 10MWT. The actuation protocol that obtained larger knee flexion than the other actuation protocols in most of the participants was M3. The M1 actuation protocol obtained larger knee flexion than F0 in participant 5, whereas the opposite situation occurred for participants 2 and 3. The pressure sensors recorded the weight distribution in the 8 sensors embedded in the shoe insole, as shown in Fig. 6. From the pressure information gathered from the shoe insoles, the CoP could be calculated. Examples from participants 3 and 5 are shown in Fig. 7. Participant 5 had an equinus foot, as shown in the CoP pattern. In contrast, participant 3 presented a CoP trajectory that covered the whole length of the shoe insole.

### Participant’s perception of the MAK

**Fig. 8:**
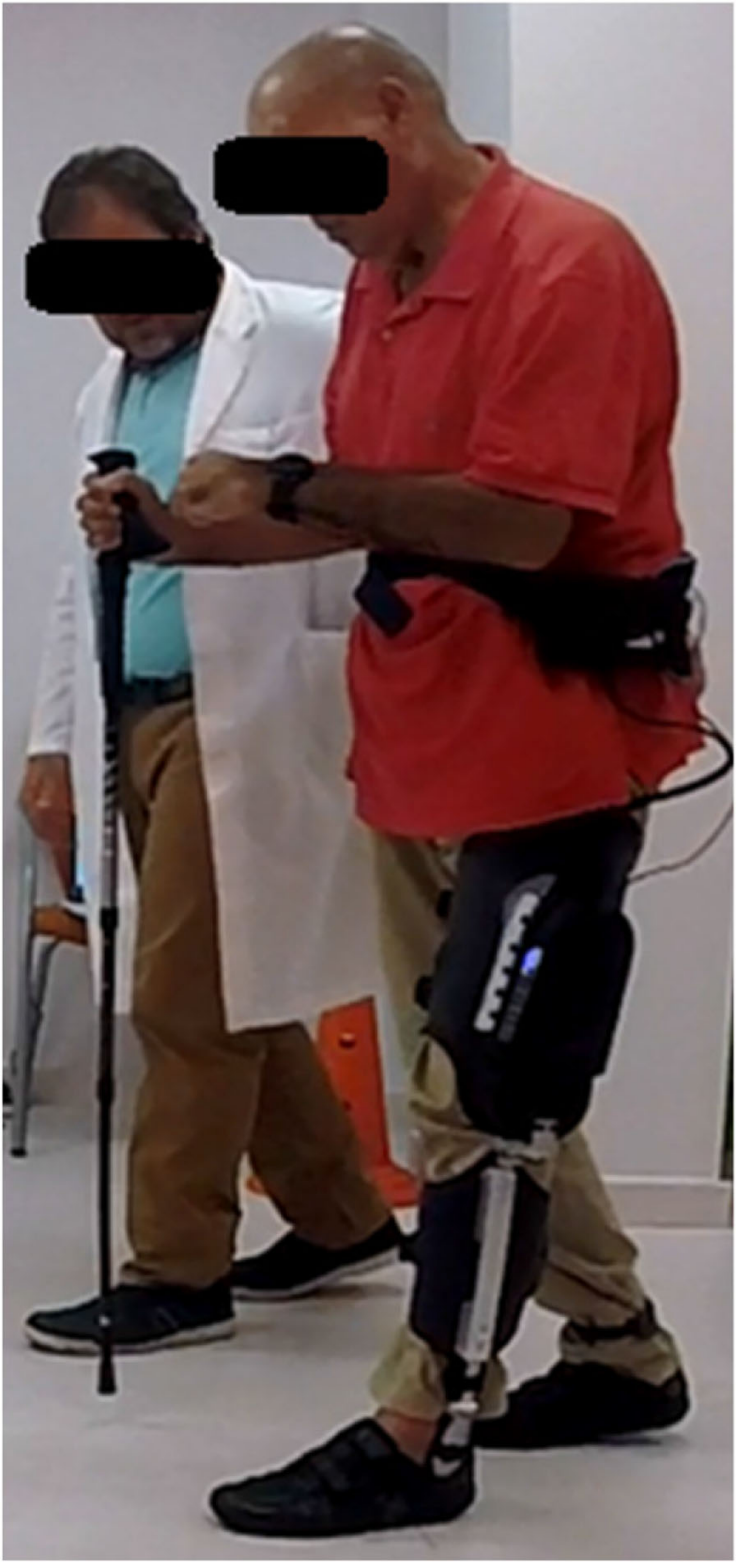
Participant using the MAK exoskeleton during a trial

The participant’s perception of the device according to the modified QUEST 2.0 scale was acceptable, and all participants were able to comfortably wear the device during all the actuation protocols (Fig. 8). The dimensions, weight, safety and security and comfort items were the highest scored items. The item with the lowest score was related to the effectiveness of the device in resolving the participant’s problems (average score 2.4 ± 0.5). According to the participants’ selection, the best features of the exoskeleton were its comfort, safety, security, and ease of use (Table 6).

**Table 6:** Modified QUEST 2.0 questionnaire scores

Item Number	Median	95% CI
**1**	3,6	0,8
**2**	2,8	1
**3**	3,6	0,5
**4**	3,6	1
**5**	2,6	0,5
**6**	2,6	1,3
**7**	3,4	1,3
**8**	2,4	0,5
**Overall Score**	2,8	0,4
**Multiple-choice**	7, 4 and 6	

QUEST 2.0 scale. Results are expressed as median and standard deviation of the values obtained. Regarding the multiple-choice question, the results are ordered from the result, which obtained more punctuation, to the lesser

## Discussion

The main objective of this study was to assess the safety and feasibility of the MAK device for use with participants with neurological conditions. Second, the MAK exoskeleton assistance actuation protocols during gait as well as the users’ perceptions about the device were assessed with clinical outcome measures. Finally, a data recording system that used the MAK sensors was tested. This is the first application of the MAK exoskeleton in participants with neurological conditions. The TRL obtained with the MAK exoskeleton based on the results of this work is 6, as it was tested in a small group of participants where the safety of the device was the main focus. Additionally, the analysis of the data gathered by the MAK made it possible to better understand the exoskeleton-user interaction to some extent.

No adverse effects were reported during this study. All participants, except for participant 1, were able to complete the study; participant 1 could not complete the study due to fatigue. Since the reason for not completing the study was not related to the device functionality, the device seems feasible and safe for use in this application, as no major adverse events were recorded. Nevertheless, an incident related to the loss of balance occurred for participant 4, and this incident was probably caused by disconnection of the shoe insole; the MAK kept functioning in M1 but did not function exactly as expected. The MAK saved the most recent data that it recorded from the last step, continued without updating these data for the rest of the trial, and remained in M1, as it was designed to do in similar situations.

The results obtained with the MAK are consistent with those of other similar commercially available devices in terms of safety and usability. Nevertheless, this comparison was made with rehabilitation-focused exoskeleton studies because to the best of our knowledge, there are no studies with the same objectives and procedures used in our study.

Previous studies conducted with a similar exoskeleton (Tibion Bionic Leg, Tibion Corporation, Sunnyvale, CA, USA) reported outcomes similar to ours. Byl et al. [43], observed no adverse events during their study, which was a case series in stroke patients. Other studies with the same device [44–46] did not report adverse events related to the use of the device. When the MAK device was used, the participants did not need any additional assistance aside from the assistance that they typically used (such as a cane or crutches). The participants could walk with the MAK device immediately since the first session involved minimal assistance; in other studies, the use of a exoskeleton with a similar amount of assistance required several training days, ranging from 4 to 10 sessions (most sessions lasted from 28 to 94 min) [47]. Nevertheless, an optimization period is probably needed to maximize the MAK assistance. Future studies should focus on assessing the time needed to optimize the assistance provided by the MAK and the time needed for the patient to get used to it. Additionally, the MAK should be updated to prevent the disconnection of insoles during its use and to include alert systems that notify the user of these situations and act accordingly.

Regarding the results obtained in the 10MWT when the user was or was not wearing the MAK, it can be observed that participants 2 and 4, who presented similar scores for the FAC and Tinetti scale, generally reported better outcomes when using the MAK than when not using the MAK. According to the G.A.I.T. scores, M3 led to slightly better scores than M1 and the condition in which the participant was not wearing the exoskeleton. M1 yielded improved G.A.I.T. scores in participants 1, 2 and 4 compared with not wearing the device, but the rest of the participants showed worse performance. This result may suggest that participants with a specific level of ambulatory independency may benefit to some extent from this device.

The differences found in the 10MWT and G.A.I.T. results may be due to the training period, as M1 can be considered a gait training mode, and M3 fully assists the gait of the user. If no training period is needed, it might be hypothesized that M3 can yield better results than the remaining actuation protocols. However, if the device is used as a rehabilitation tool, M1 can enhance the patient’s gait ability due to the algorithm design, as it needs more participation from the user to provide assistance. Additionally, these differences in the results may be due to the actuation protocol designs. As M3 does not need the participant to exert forces at the knee, it may detect changes in the gait cycle sooner than M1 because the participants showed some level of spasticity with M1. The analysis of the results obtained with M1 shows that the data from participant 4 should be interpreted with caution because the actuation protocol M1 did not provide the same level of assistance as it did for the rest of participants due to the insole being disconnected. Future studies should focus on assessing possible gait improvements due to the use of M1 and measure the differences between conditions in which the MAK is used with objective measurement systems.

The gait speed achieved by the participants during the use of the MAK device was similar to previously reported results in patients with stroke. Beyaert et al. [48] reported a range of gait speed in stroke patients from 0,23 ± 0,11 to 0,73 ± 0,38 m/s. The mean gait speed obtained in the present study was 0,39 ± 0,21 m/s while F0 was applied, 0,36 ± 0,18 m/s while M1 was used and 0,53 ± 0,13 m/s while M3 was implemented. The values obtained while using the MAK are distant from the healthy gait speed observed by Beauchet et al. [49], which is 1,25 ± 0,22 m/s.

Additionally, the MAK recorded data related to the knee joint angle, pressure at the insole and the CoP. According to knee joint angles, every participant who used M3 seemed to have improved knee flexion angles. This result may reflect an improvement in the gait pattern due to an equinus gait used to prevent falls. Determining the CoP, joint angle and plantar pressure at each point of the shoe insole may help rehabilitation teams better assess the progression of equinus foot recovery, for example, and improve the effectiveness of therapy for the participant. Future studies must be conducted to validate the measures recorded by the MAK device.

According to the results obtained in the modified QUEST 2.0 questionnaire, the MAK presented an acceptable perceived use (overall average score of 2.8 ± 0.4 95% CI out of 5). According to the users’ perspectives, the strengths of the device were its size, safety, ease of adjustment, weight and comfort. The item with the lowest score was related to the effectiveness of the device in resolving the participant’s problems. This result might be due to the short training period that the participants received and may be related to the participant’s expectations. Kozlowski et al. [50], in a study using the ReWalk™ in MS participants, obtained a mean score of 3.7 in the QUEST 2.0 questionnaire. These results may be linked to an extended period in which the user wore the exoskeleton, which may lead the scores for items such as effectiveness to be rated higher.

This study presents several limitations. First, the number of participants was limited, and therefore, the results cannot be generalized to individuals with other neurological diseases or all individuals with stroke and MS. Since the insole with embedded sensors placed under the foot was eventually disconnected for participant 4, the corresponding results regarding the use of the M1 should not be considered representative. The data presented in this paper are descriptive, as this is a proof-of-concept study, and can serve as guidelines for future clinical studies investigating the clinical effects of MAK on gait assistance. To better assess the effectiveness, additional studies with improved measurement instruments will be carried out. Finally, the period of adaptation to the MAK was rather short (only 5 sessions per participant prior to the beginning of this study, with short session durations), which may have impacted the results.

## Conclusions

All recruited participants were able to use the MAK exoskeleton successfully without any adverse effects, but an incident related to the loss of stability (without falls or any other major implications) was detected. The results obtained in this proof-of-concept study may suggest that certain actuation protocols for the device can improve the gait performance of stroke and MS patients. The MAK exoskeleton seemed to afford positive preliminary results with respect to its safety, feasibility, and user acceptance. The efficacy of the MAK should be studied in future studies, and more advanced improvements should be implemented to prevent unexpected device behaviors.

## Data Availability

All the data and materials could be found at MARSI BIONICS.

## Abbreviations

10MWT: 10-m walking test
AFO: Ankle-foot orthosis
CoP: Center of Pressure
DALYs: Disability-adjusted life-years
FAC: Functional Ambulation Category
G.A.I.T.: Gait Assessment and Intervention Tool
MAK: Marsi Active Knee
M1: Modes of action 1
M3: Modes of action 3
MAS: Modified Ashworth Scale
MS: Multiple sclerosis
QUEST 2.0: Quebec User Evaluation of Satisfaction with assistive Technology test
TRL: Technology Readiness Level
F0: Zero force control mode
